# A 3-Month Modified Basketball Exercise Program as a Health-Enhancing Sport Activity for Middle-Aged Individuals

**DOI:** 10.3390/life14060709

**Published:** 2024-05-30

**Authors:** Konstantina Karatrantou, Konstantinos Pappas, Christos Batatolis, Panagiotis Ioakimidis, Vassilis Gerodimos

**Affiliations:** Department of Physical Education and Sports Science, University of Thessaly, 42100 Trikala, Greece; kokaratr@uth.gr (K.K.); ntinospappas7@gmail.com (K.P.); batatoli@uth.gr (C.B.); ioakeimidis@uth.gr (P.I.)

**Keywords:** team sports, functional capacity, physical fitness, combined training, enjoyment

## Abstract

Recreational team sports have received great acceptance lately, in different populations, indicating encouraging results in health-related quality of life. This study examined the efficacy of a 3-month basketball exercise program on selected indices of health (body mass—BM, body fat—BF, blood pressure—BP), functional capacity (flexibility of lower and upper limbs, balance), and physical fitness (maximum strength of lower limbs, trunk and handgrip, aerobic capacity) in middle-aged individuals. Forty middle-aged individuals (males and females; 40–55 years old) were randomly divided into (a) exercise (EG; *n* = 20) and (b) control groups (CG; *n* = 20). The EG followed a 3-month modified basketball exercise program (2 times/week; 24 training units), including different basketball drills with and without the ball (dribbling, passing, pivot, stops, etc.), to improve participants’ health and physical fitness. Repeated measures ANOVA showed that the EG significantly increased their flexibility (17.23–74.88%; *p* < 0.001), static balance (44.76–54.69%; *p* < 0.001), and strength of lower limbs and trunk (11.67–13.13%; *p* < 0.001), while reducing BP (7.31–12%; *p* < 0.001), heart rate and RPE (5.30–34.37%; *p* < 0.001), and time during time-up-and-go test (−10.91%; *p* < 0.001). Handgrip strength, BM, and BF did not change following the program in the EG (*p* > 0.05). In the CG, the above variables remained stable. In conclusion, this program may be used to eliminate the detrimental effects of aging on health, functional capacity, and physical fitness parameters.

## 1. Introduction

In recent decades, there has been a significant increase in the population of middle-aged individuals (40–64 years old), where they represent approximately 25% (1.98 billion inhabitants) of the total global population (7.95 billion inhabitants) [1,2]. Middle age is a crucial phase of the human lifespan, which is characterized by significant and rapid changes in physical, mental, cognitive, and social domains [3,4]. These changes, in conjunction with reduced physical activity levels and the adoption of unhealthy behaviors, are considered important risk factors that are related to the development of different chronic diseases (obesity, hypertension, cardiovascular diseases, diabetes, etc.), the reduction in self-care capacity (independent living), and the degradation of quality of life [5,6]. Taking all the above into consideration, the World Health Organization (Global Action Plan on Physical Activity 2018–2030) and the European Union (Health-enhancing physical activity policy) promote active aging using different exercise interventions, so that middle-aged individuals remain healthy for a longer period and continue to contribute to the labor market and society in general [7,8]. Lately, recreational team sports have gained popularity, offering an alternative form of exercise for health promotion across the lifespan and sexes [9,10,11,12].

Participation in different recreational team sports, apart from the positive impact on health, functional capacity, and physical fitness parameters [9,10,11,12] that is equally comparable with other more traditional forms of exercise, promotes several life skills, such as co-operation, teamwork, respect for yourself and others, communication, discipline, and perseverance. In the scientific literature, several studies examined the efficacy of different team sports programs, using soccer/football [12,13,14,15], handball [16,17,18,19,20,21,22,23,24], basketball [25,26,27] or streetball (street basketball) [28], floorball [29,30,31,32], netball [33,34], volleyball [35,36], rugby [37,38,39] and futsal [40,41], demonstrating promising results in selected physical, mental, and social indicators of healthy individuals as well as of individuals with diseases. However, it should be mentioned that the vast majority of the aforementioned studies (a) focused on soccer/football and handball, while there is limited information on other team sports (including basketball), (b) used a competitive approach during their training (using small-sided games 3 vs. 3, 5 vs. 5), without focusing on the modification of team sports exercises and drills for the enhancement of specific fitness parameters, and (c) focused on the evaluation of isolated or selected physical fitness parameters.

One of the most widespread team sports all over the world is basketball, with approximately 2.2 billion fans and 450 million players worldwide (approximately 100 million are recreational players) [10,42,43]. Basketball as a team sport has several advantages, such as (a) easy access (in indoor and outdoor spaces), (b) low-cost equipment, (c) easy exercise/skill modification according to the population characteristics, and (d) multifaceted involvement of the whole body (lower and upper body) in various skills and movements. The few studies examining the efficacy of a sole basketball intervention program were performed in young healthy adults [28,44,45,46,47] as well as in children and adolescents with autism, intellectual disabilities, etc. [48,49,50,51,52,53] or young adults/college students with Down syndrome, intellectual disabilities, obesity, etc. [25,26,27], while no previous study focused on middle-aged or older individuals. To provide more detail, these previous studies showed that basketball training improves different functional capacity and physical fitness parameters (flexibility, balance, strength and/or aerobic capacity) in young, healthy adults as well as in young adults, children, and adolescents with autism, intellectual disabilities, etc., while the results regarding body composition and blood pressure are conflicting (either reduction or no effect) [25,26,27,28,44,45,46,47,48,49,50,51,52,53]. Furthermore, previous studies showed that basketball training causes significant positive effects on mental health, decreasing feelings of anxiety, loneliness, inadequacy, and perceived stress [46], and increasing self-respect, belief value, and role adaptation in college students [47]. However, it should be noted that several of the aforementioned studies in basketball focused solely on the evaluation of social and mental parameters [44,46,47,48,49,50], whereas most of the few studies that examined physiological parameters did not use a comprehensive physical fitness profile (flexibility, balance, strength, and aerobic capacity). The only study [25] that implemented a modified basketball exercise program for improvement in overall physical fitness has been performed in young adults with Down syndrome and showed promising results in all measured parameters (hip and waist circumference, body mass, body mass index, flexibility, strength, aerobic capacity, balance, and basketball functional ability).

Thus, the main objective of this research was to design, implement, and evaluate, for the first time, a 3-month modified basketball exercise program in middle-aged males and females, aiming to improve selected health, functional capacity, and physical fitness parameters. Participants’ enjoyment following the basketball exercise program was also investigated. We hypothesized that the 3-month modified basketball exercise program (2 times/week) would be an enjoyable sport activity that would improve all health, functional capacity, and physical fitness parameters.

## 2. Materials and Methods

### 2.1. Participants

In the present study, initially, 50 healthy middle-aged individuals (males and females), from the region of Thessaly, Greece, were recruited to participate in this study. Before commencement of the study, a power analysis, using software package GPower 3.1, for statistical test “ANOVA: Repeated measures, within-between interaction (2 groups and 2 measures)” and small effect size 0.25 was performed. The statistical power analysis indicated that a total of 40 participants (20 participants in each group) would yield adequate power (0.868).

Before the initiation of the study, the participants were checked according to the screening criteria. In more detail, the participants (a) had to be in the middle age category (40–64 years old), (b) should not have suffered from any chronic disease, illness, or injury (as this was assessed using the American College of Sports Medicine (ACSM) health history questionnaire) [54], and (c) should not have received the use of any medication. Furthermore, they should not have participated in regular organized physical activity for at least 6 months before the study and should not have followed any dietary plan (supervised or unsupervised) to reduce body mass and body fat.

After checking the screening criteria, 10 participants were excluded. Thus, the final sample of the study was 40 middle-aged males and females, who were randomly divided into two equal groups: (a) exercise group (*n* = 20) and (b) control group (*n* = 20).

Before the start of the study, the participants were analytically informed by the investigator regarding the aim, the experimental procedures, and the possible risks during the study, and signed an informed consent form. The study was conducted according to the Declaration of Helsinki and the Ethics Committee of the University of Thessaly at Greece granted the ethical approval.

### 2.2. Study Design—Procedure

Before the start of the study, participants were informed about the research and were familiarized with the basic basketball skills as well as with the measurements. Thereafter, baseline measurements were performed on two consecutive days for each participant. On the first day, anthropometric characteristics (body mass and height), blood pressure, body composition, flexibility, and balance were measured, while on the second day, handgrip strength, maximum lower limb and trunk strength, as well as aerobic capacity, were measured. Following the baseline measurements, the participants were randomly assigned to two equal groups: the EG and the CG. A computer-generated list of random numbers was used for the allocation of the sample in one of the two groups. During the study, the EG participated in a 3-month (12 weeks; 2 times/week; 24 training units) modified basketball exercise program, while the CG was asked not to engage in any exercise during the 3 months. The no-participation of the CG in exercise programs were monitored before and after the time period using the Health History Questionnaire of the ACSM [54]. It would be important to mention that no adverse effects or injuries were reported during the 3-month exercise program. An exercise instructor with a specialization in basketball supervised the program. After the end of the exercise program, the baseline measurements were repeated in the same order, at the same time of day, and under the same conditions. All the measurements and the exercise program were performed at an indoor basketball court.

### 2.3. Exercise Program

During the study, EG attended a 3-month modified basketball exercise program, with 2 training units per week (a total of 24 training units), in contrast to the control group, which did not follow an exercise program. Each training unit lasted 55–65 min, including three parts: (a) warm up (12–18 min; 5 min modified basketball drills with movement and 7–13 min static and dynamic stretching exercises for the whole body), (b) main part (35–45 min; modified basketball drills for physical fitness enhancement), and (c) recovery (8–10 min; walking on the basketball court and static stretching for the whole body). The main part of the exercise program included modified basketball exercises and drills with (passing, dribbling, shooting, etc.) and without the ball (pivot, stops, sliding, etc.) to improve specific indices of physical fitness (Table 1). All training contents and training variables were gradually changed/increased during the 3-month exercise program.

**Figure 1:**
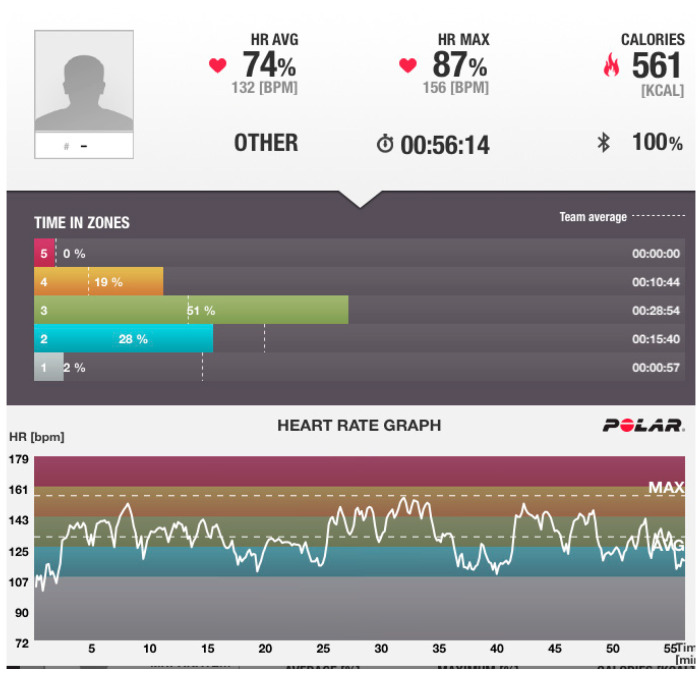
Individual report of an indicative daily heart rate recording.

During the exercise program, a real-time monitoring of the participant’s heart rate using the system Polar Team Solution (Science Technologies, Kempele, Finland) was performed. The report from an individual indicative daily heart rate recording during the exercise program is graphically presented in Figure 1.

An indicative training unit of the 3-month modified basketball exercise program is analytically presented in Table 2.

### 2.4. Measurements

#### 2.4.1. Health, Functional Capacity, and Physical Fitness Parameters

Before and immediately after the end of the exercise program, selected health, functional capacity, and physical fitness parameters were evaluated using worldwide-used and reliable testing protocols for middle-aged individuals [54,55,56,57,58,59,60] (Table 3). During the measurements, a standardized 10-min warm-up (low-intensity running, static and dynamic stretching exercises for the whole body) and 5-min recovery were performed (static stretching for the whole body).

#### 2.4.2. Enjoyment

The subscale of the intrinsic motivation questionnaire of Mc Auleys et al. [61] was used to assess the EG participants’ enjoyment following the 3-month modified basketball exercise program. The subscale for enjoyment assessment included 4 questions: 1. I enjoyed the activities of the program, 2. I thought that the program was interesting, 3. I thought that the time passed very quickly when I participated in the program, and 4. When I participated in the program, I enjoyed it [61]. The questions were scored using a 5-point Likert scale according to the level of agreement or disagreement of the participants in each question. The scale of response for all the questions was as follows: 1: strongly disagree, 2: disagree, 3: neither agree nor disagree, 4: agree, and 5: strongly agree. The scores 4 and 5 denote good and high levels of enjoyment following an intervention program. The score of each question as well as the total score from all questions were considered for analysis.

### 2.5. Statistical Analysis

The data were analyzed using IBM SPSS Statistics (IBM Corp. Released 2019. IBM SPSS Statistics for Windows, Version 26.0. Armonk, NY, USA: IBM Corp.). For each of the variables, an adjustment test to a normal distribution was performed using the Shapiro–Wilk criterion (all variables followed the normal distribution). To examine the effect of the 3-month modified basketball exercise program on health, functional capacity, and physical fitness parameters, repeated measures analyses of variance “group” × “time” (2 × 2) were used, with repeated measurements on the factor “time”. In addition, Sidak analysis was used to investigate differences between groups where necessary. Independent *t*-tests were used to compare the percentage changes from baseline to final measurements in all tested parameters between groups (EG vs. CG). Independent *t*-tests were also used to compare demographic and anthropometric characteristics between EG and CG. The significance level was set to *p* < 0.05.

## 3. Results

The sample of the study consisted of 40 middle-aged males and females (aged 40–55 years old), who were randomly divided into two equal groups: (a) an exercise group (*n* = 20) and (b) a control group (*n* = 20). In each group, there was an equal number of males and females. The demographic and anthropometric characteristics of participants are analytically presented in Table 4.

Independent *t*-tests, before the start of the study, showed no significant differences (*p* > 0.05) on demographic and anthropometric characteristics between the two groups (Table 4).

### 3.1. Health Parameters

Repeated measures analyses of variance showed a statistically significant interaction effect in blood pressure (systolic and diastolic) (*p* < 0.001), in contrast to body fat and body mass, in which there were no statistically significant interactions or simple effects (*p* = 0.828–0.896). In the EG, systolic and diastolic blood pressure values following the exercise program were significantly lower than before the implementation of the program (*p* < 0.001), while in the CG they remained unchanged (*p* > 0.05). Blood pressure values before the exercise program did not differ between the two groups (*p* > 0.05), while blood pressure values following the exercise program were significantly lower in EG compared to CG (*p* < 0.001; Table 5). The percentage changes (from baseline to final measurements) in blood pressure (systolic and diastolic) were significantly greater in the EG than in the CG (*p* < 0.001). Regarding body fat and body mass, no significant changes were observed between groups and measurements in both EG and CG (*p* > 0.05).

### 3.2. Functional Capacity Parameters

Repeated measures analyses of variance showed a statistically significant interaction effect in flexibility of lower and upper limbs as well as in static and dynamic balance (*p* < 0.001). In the EG, the final flexibility and static balance values were significantly higher than the respective baseline values (*p* < 0.001), while the final values of dynamic balance (time in sec during the time up and go test) were significantly lower than the corresponding baseline values (*p* < 0.001; Table 6). In the CG, all the above functional capacity parameters remained unchanged over the 3 months (*p* > 0.05). The functional capacity parameters before the implementation of the exercise program did not differ between groups (*p* > 0.05), whereas following the program, there were significant differences between the EG and CG in all measured parameters (*p* < 0.001). The percentage changes (from baseline to final measurements) in functional capacity parameters (flexibility and balance) were significantly greater in the EG than in the CG (*p* < 0.001).

### 3.3. Physical Fitness Parameters

#### 3.3.1. Strength

Repeated measures analyses of variance showed a statistically significant interaction effect in maximum strength of lower limbs and trunk (*p* < 0.001), in contrast to handgrip strength where no statistically significant interaction was demonstrated (*p* > 0.05). In the EG, lower limbs’ and trunks’ strength values following the exercise program were significantly higher than before the exercise program (*p* < 0.001), while handgrip strength values remained unchanged (*p* > 0.05; Table 7). In the CG, all of the above physical fitness indicators remained unchanged during the 3 months (*p* > 0.05). Baseline strength values of lower limbs and trunk did not differ between the two groups (*p* > 0.05), whereas following the exercise program, these values were significantly higher in the EG compared to the CG (*p* < 0.001). The percentage changes (from baseline to final measurements) in maximum strength of lower limbs and trunk were significantly greater in the EG than in the CG (*p* < 0.001).

#### 3.3.2. Aerobic Capacity

Repeated measures analyses of variance showed statistically significant interactions in all the aerobic capacity parameters (*p* < 0.001). In the EG, heart rate and rate of perceived exertion (RPE) values (using the 10-point Borg scale), following the exercise program, were significantly lower than before the intervention program (*p* < 0.05–0.01; Table 8). In the CG, the above variables remained unchanged during the 3 months (*p* > 0.05). Baseline heart rate and RPE values did not differ between the two groups (*p* > 0.05), whereas following the exercise program, these values were significantly lower in the EG compared to the CG (*p* < 0.001). The percentage changes (from baseline to final measurements) in heart rate and RPE were significantly greater in the EG than in the CG (*p* < 0.001).

### 3.4. Enjoyment

According to the results of the study, the participants in the EG demonstrated good or high levels of enjoyment following the 3-month modified basketball exercise program. In more detail, 80% of the EG participants reported high levels of enjoyment (score 5 in the 5-point Likert scale) in all the questions of enjoyment, while 20% of the EG participants (2 males and 2 females) reported good or high levels of enjoyment (score 4 or 5 in the 5-point Likert scale). The mean as well as the minimum and maximum score of the participants in each question is analytically presented in Table 9.

## 4. Discussion

The novel aspect of this study lies in the design, implementation, and evaluation of the efficiency of a 3-month modified basketball exercise program on selected health, functional capacity, and physical fitness indicators of middle-aged individuals. As we mentioned earlier, to date, the few studies that examined the effect of a solely basketball-based intervention program have been performed in children or young adults. Our results demonstrated that a 3-month (2 days/week; 24 training units in total) modified basketball exercise program is an enjoyable intervention that reduces blood pressure and induces significant improvements in middle-aged individuals’ flexibility, balance, strength, and aerobic capacity, thus proving our research hypothesis. On the other hand, it seems that it does not affect body mass, body fat, and handgrip strength, disproving our research hypothesis on these parameters.

One of the most important findings of this study is the significant reduction in systolic (8.3 mmHg; 7.3%) and diastolic (9 mmHg; 12%) blood pressures following the intervention program. According to the American College of Cardiology and the American Heart Association, normal systolic and diastolic blood pressure values are lower than 120 and 80 mmHg, respectively [62]. The reduction in blood pressure (to more normal values) following a systematic exercise intervention may eliminate the risk of cardiovascular diseases (i.e., coronary heart disease, cerebrovascular disease, rheumatic heart disease, and other conditions), which are the leading cause of death globally (taking approximately 17.9 million deaths each year) [63]. Several previous studies have shown that different recreational team-sport activities (i.e., football, floorball) [29,64,65] and other combined exercise programs using various modes of exercise [66,67,68,69,70,71] effectively decrease blood pressure (systolic and diastolic) in middle-aged individuals, supporting the results of the present study. Regarding the effect of recreational basketball training on blood pressure, there is limited information in the scientific literature with conflicting results. The findings of this study partially agree with those of Randers et al. [28], who demonstrated that 3 months of supervised recreational street basketball training (using 3 vs. 3 games on a full court with 2 baskets) significantly reduced systolic blood pressure in untrained men, while diastolic blood pressure did not change, in contrast to our study. Furthermore, previous studies found that 3-month supervised recreational basketball training (using 3 vs. 3 games on a half-court with 1 basket) [28] or 12 weeks of supervised recreational basketball training with small-sided games (3 vs. 3 games on half-court) [45] did not change blood pressure in young adults. The conflicting results between our study and previous studies may be attributed to different factors such as the participants’ chronological age (28.4 ± 7.0 years old in the Randers et al. study and 19.63 ± 0.67 years old in the Rakesh et al. study vs. 46.15 ± 4.37 years old in the present study), the baseline levels of blood pressure, as well as the training characteristics. Our participants had higher baseline systolic (122.4 ± 15 mmHg) and diastolic blood pressure (84 ± 10.10 mmHg) levels than the participants of Rakesh et al. [45] (systolic: 114.18 ± 7.18 mmHg and diastolic: 71.72 ± 6.31 mmHg), which may account for the divergent results.

It should also be mentioned that the 3-month modified basketball exercise program of the present study significantly improved different features of functional capacity and physical fitness such as flexibility of lower and upper limbs (17.2–74.88%), static (44.76–54.69%) and dynamic balance (10.91%), maximum strength of lower limbs and trunk (11.67–13.13%), as well as aerobic capacity. Our findings are in line with those of few previous studies that performed recreational basketball training (12–24 weeks; 2–3 times/week), using either small side games (3 vs. 3) or modified exercise programs with basketball drills, and found significant improvements in flexibility [25], balance [25], strength [25,26] and aerobic capacity [25,28,45] in young healthy adults or those with autism, intellectual disabilities, etc. Basketball is a team sport that is influenced by both anaerobic and aerobic metabolism [72,73,74]. On the one hand, basketball is considered an intermittent high-intensity sport consisting of different skills such as shooting, jumping, blocking, passing, and lay-ups that require mainly the contribution of anaerobic metabolism [75,76,77], leading therefore to significant improvement in different physical fitness parameters (i.e., strength, power). In our study, we designed and implemented a modified basketball exercise program consisting of exercises and drills with (passing, dribbling, shooting, etc.) and without the ball (pivot, stops, sliding, etc.) to improve specific indices of physical fitness (strength, power, balance etc.). The basketball drills that we used multifaceted involvement of the whole body, which explains the improvement in both lower and upper body strength. In the same context, previous studies demonstrated that basketball training causes significant improvement in maximal isokinetic (concentric and eccentric) and isometric strength of lower limbs in adults with intellectual disabilities [26], in the endurance strength of abdominal muscles, and in the power of lower limbs in adults with Down syndrome [25]. On the other hand, basketball (due to the duration of game: 40–48 min) requires a high level of aerobic metabolism to enhance the resynthesis of creatine phosphate, lactate clearance from active muscle and removal of accumulated intracellular inorganic phosphate [78], leading potentially to significant improvement in aerobic capacity. In our study, we used a 3-month modified basketball exercise program with total duration per training unit of 55–65 min that caused a significant improvement in participants’ aerobic capacity. Previous studies also showed significant improvement in aerobic capacity following basketball or street basketball (streetball) training in adults with Down syndrome [25], in adolescents with intellectual disabilities [52,53] and in healthy adults [28,45]. Furthermore, the results of the present study are comparable with other studies, which used different recreational team sport interventions (soccer/football, handball, netball) [13,14,16,17,22,33] as well different other exercise activities/programs (i.e., combined exercise programs, Zumba, resistance–functional training) [13,14,67,68,69,70,71] and found significant improvements in various physical fitness parameters of healthy middle-aged and older individuals.

In the present study, the only physical fitness parameter that did not change following the intervention program is maximum handgrip strength. Although basketball involves various skills that rely on the continuous use of wrist and digits flexors muscles in catching, holding, shooting, and throwing the ball [79,80], it does not involve squeeze movements that simulate handgrip strength measurement. However, handgrip strength is an important indicator of hand function and overall functional capacity [81]. Reduced handgrip strength is associated with functional decline and disability during daily activities and future morbidity/mortality in middle-aged and older individuals [82]. From the results of this study, it seems that a more specialized exercise program is needed. Several previous studies that examined the effectiveness of different specialized strength training programs (especially for forearm, wrist, and fingers) using different training means (i.e., elastic balls, hand grippers, therapeutic clay) reported significant improvements in maximal and endurance handgrip strength of young, middle-aged, and older healthy individuals as well as individuals with autism, intellectual disabilities, etc. [67,68,69,71,83,84,85,86].

Although it is well known that regular participation in physical activities is associated with significant physical and mental health improvements, many individuals drop out of exercise programs due to the lack of enjoyment and monotony of exercise [87,88]. One of the most important strategies to increase exercise adherence and avoid dropouts is the promotion of positive feelings and satisfaction during the exercise program [87,88]. Participation in team sports interventions is an ideal choice because, beyond the physical and mental benefits, it improves teamwork, communication, and collaboration factors that could positively affect satisfaction and enjoyment during exercise [10]. In our study, we found that a 3-month modified basketball exercise program causes high levels of enjoyment in middle-aged males and/or females.

This study has some limitations that could affect the generalization of its findings. First, the results of this study are limited to middle-aged individuals and to the use of a 3-month (12 weeks; 2 times/week; 24 training sessions in total) modified basketball exercise program. Future studies could examine the efficacy of basketball intervention programs with different training characteristics or could examine the efficacy of a modified basketball exercise program in elderly individuals. It should be mentioned that the present study used a mixed sample of males and females (with a wide performance range in functional and physical fitness parameters), something that could affect the results of the study. Upcoming studies could compare the training adaptations between males and females following a basketball exercise intervention for health promotion. Finally, another limitation of this study is the frequency of training (2 times/week), which nevertheless caused significant improvements in selected functional and physical fitness parameters. The use of greater training frequency per week (>2 times/week) could give even greater benefits by improving all health, functional capacity, and physical fitness parameters.

In conclusion, a 3-month modified basketball exercise program using drills with and without the ball (specifically designed to improve flexibility, balance, strength, and aerobic capacity), even with a frequency of 2 times/week, significantly improved different features of health, functional capacity, and physical fitness in middle-aged males and females. This program is an enjoyable physical activity that could be safely used to reduce the harmful effects of aging on health, functional capacity, and physical fitness parameters.

## Figures and Tables

**Table 1:** Training contents and variables of the exercise program.

Training Goals	Training Contents	Training Variables/Methods
Flexibility	✓ Static stretching for the whole body. ✓ Dynamic exercises for the whole body.	Set: 1–2. Duration/Repetitions per set: 10–20 s (for static stretching)/10–20 reps (for dynamic exercises).
Balance	Static: ✓ Single-leg balance with the ball (toss and catch the ball in the air with a clap, dribbling, passing). ✓ Control dribbling in pairs while the teammate pushes the body in all directions. Dynamic: ✓ Running and stopping in defensive and basic stances. ✓ Forward dribbling with jump and stride stops and pivots. ✓ Forward running–sudden stop–backpedaling–forward running.	Set: 1–3. Distance/Duration per set: 10–20 m (in exercises with movement)/10–25 s (in static balance exercises).
Strength	Stationary Dribbling Drills: ✓ Control dribbling in high and low positions. ✓ Control dribbling in pairs while the teammate pushes the body in all directions. Moving Dribbling Drills: ✓ Forward dribbling with jump and stride stops and pivots. ✓ Forward dribbling–sudden stop–backward dribbling–forward dribbling. ✓ Forward dribbling with changes in directions. Passing Drills: ✓ Chest, bounce, overhead passes. Combined drills ✓ Control dribbling-forward dribbling–passing–returning with backpedaling or lateral steps or defensive slides. ✓ Drills with jump-stop and jump-shot in the basket.	Set: 1–3. Distance/Duration/Repetitions per set: 10–20 m (in exercises with movement)/20–30 s (in static exercises)/10–20 reps.
Aerobic capacity	Combined drills or games: ✓ Basketball drills with running, lateral steps or defensive slides, backpedaling, and change in direction. ✓ Competitive team games with basketball drills (dribbling, passing, shots).	Training method: Interval and Continuous. Total duration: 14–20 min. Intensity: 65–85% of age-predicted maximum heart rate.

**Table 2:** Indicative training unit (13th training unit) of the exercise program.

	Total Duration 63 min
Warm-up (18 min)	1st Exercise In pairs opposite each other on the two sidelines. ✓ Control dribbling with bounces (10 dribbles), forward dribbling, chest pass, and returning with backpedaling (2 sets/dribbling hand). ✓ Single leg balance and control dribbling, forward dribbling, bounce pass, and returning with defensive slides (2 sets/leg). 2nd Exercise Dynamic stretching. ✓ Upper body static and dynamic stretching exercises during walking. ✓ Lower body stretching exercises.
Main part (35 min)	3rd Exercise Chest, bounce, and overhead passes in pairs (×45 total). 4th Exercise Foul court routes on cones, ending with jump-shot (2 teams). ✓ Reaching each cone, perform 10 control dribbles with high intensity (×2 reps). ✓ Reaching each cone, doing some backward dribbling, and moving forward to the next cone (×2 reps). ✓ Reaching each cone, perform 2 control dribbles with high intensity and crossover dribble (×2 reps). ✓ Performing the same drills with the other hand. 5th Exercise Competitive game (4 teams), 3 min × 2 sets with 30 s rest with walking. One team at each corner of the court. The first player performs forward dribbling until the middle of the court and returns to the basket and performs a jump shot. To count the basket, the player should return to the middle of the court and shout the team’s score. The team with the highest score wins.
Recovery (10 min)	Walking two courts. Static stretching of the whole body.

**Table 3:** Health, functional capacity, and physical fitness measurements before and after the exercise program.

Health Parameters	
✓ Body mass was assessed using a calibrated physician’s scale (Seca model 755, Seca, Hamburg, Germany), as previously described by ACSM [54]. ✓ Percentage of body fat (%BF) was assessed using the bioelectrical impedance method (Maltron 900), as previously described by ACSM [54]. ✓ Blood pressure (systolic and diastolic) was assessed using an electronic upper arm blood pressure monitor (A&D-UA-851), as previously described by ACSM [54].	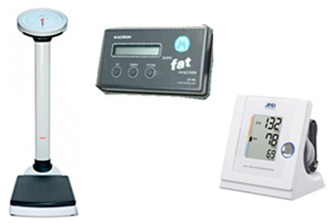
Functional capacity parameters	
Flexibility ✓ Lower back and hamstring flexibility was assessed with the sit-and-reach test using a Flex-Tester box (Novel Products Inc., Rockton, IL, USA), as previously described by ACSM [54]. ✓ Shoulder range of motion was assessed with the back scratch test using a measuring tape, as previously described by Corbin et al. [56]. The participants performed 3 maximal trials at each test, and the best score in cm was considered for analysis. Balance ✓ Static balance was assessed for both legs using the single-limb stance test with eyes open, as previously described by Rinne et al. [57] (3 trials/leg, the average time in sec was considered for analysis). ✓ Dynamic balance was evaluated using the timed up-and-go test (TUG), as previously described by Rikli and Jones [58] (3 trials; the best time in sec was used to evaluate performance).	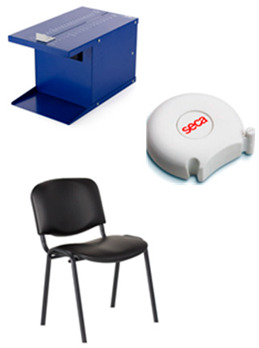
Physical fitness parameters	
Strength ✓ Maximal isometric handgrip strength was assessed using a portable hydraulic dynamometer (Jamar 5030J1, Horsham, USA), as previously described by Ruiz et al. [59] and Karatrantou and Gerodimos [55]. The best score (in kg) of 3 isometric contractions (1 min rest/trial), at each hand, was considered for analysis. ✓ Maximum trunk and leg strength were measured, with a portable Takei back and leg dynamometer (Takei, Analogue dynamometer 5002, Japan), as previously described by Coldwells et al. [60] and Karatrantou and Gerodimos [55]. The participants performed 3 maximal trials at each test (1 min rest/trial) and the best score (in kg) at each test was considered for analysis. Aerobic capacity The YMCA 3-min step test (30 cm box) was used to assess aerobic capacity (metronome cadence: 96 beats per minute; 4 steps per cycle), per Karatrantou and Gerodimos [55]. Participants’ (a) heart rate (HR) before the test, (b) HR and rate of perceived exertion using the Borg scale at the end of the test, and (c) HR 1 min following the termination of the step test were measured and considered for analysis.	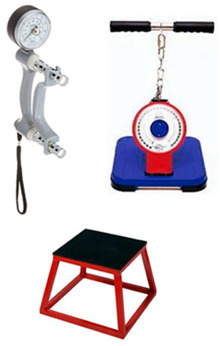

**Table 4:** Demographic and anthropometric characteristics of participants in the exercise (EG) and control groups (CG).

	EG (*n* = 20)	CG (*n* = 20)
Variables		
Age (years)	46.15 ± 4.37	46.20 ± 3.67
Sex	10 males, 10 females	10 males, 10 females
Body height (m)	1.70 ± 0.12	1.71 ± 0.14
Body mass (kg)	80.22 ± 17.71	81.30 ± 18.50
Body mass index (kg/m^2^)	27.06 ± 4.29	27.74 ± 5.00

**Table 5:** Health parameters in exercise and control groups per measurement (Mean ± Standard deviation).

Variables	Group	Baseline Measurement	Final Measurement	Interaction Effect	Mean % Change
Body mass (kg)	EG CG	80.22 ± 17.71 81.30 ± 18.50	80.16 ± 17.75 81.20 ± 18.00	*F*_1,38_ = 1.65, *p* = 0.896	−0.07 −0.12
Body fat (%)	EG CG	29.93 ± 6.12 29.96 ± 7.40	29.73 ± 6.59 29.85 ± 7.40	*F*_1,38_ = 1.78, *p* = 0.828	−0.67 −0.37
BP Systolic (mmHg)	EG CG	122.40 ± 15.00 123.00 ± 18.00	114.10 ± 11.00 *# 124.00 ± 15.00	*F*_1,38_ = 22.00, *p* < 0.001	−7.31 † +0.81
BP Diastolic (mmHg)	EG CG	84.00 ± 10.10 85.00 ± 12.00	75.00 ± 9.50 *# 84.00 ± 10.00	*F*_1,38_ = 25.56, *p* < 0.001	−12 † −1.19

BP: blood pressure; CG: control group; EG: exercise group. Where * *p* < 0.001, there is a statistically significant difference between baseline and final measurements in the EG; # *p* < 0.001 = statistically significant difference between the EG and CG in the final measurement; † *p* < 0.001 = statistically significant difference between the EG and CG in the percentage change.

**Table 6:** Functional capacity parameters in exercise and control groups per measurement (Mean ± Standard deviation).

Variables	Group	Baseline Measurement	Final Measurement	Interaction Effect	Mean % Change
Flexibility					
Sit-and-reach test (cm)	EG CG	13.24 ± 11.22 13.50 ± 10.00	16.68 ± 10.99 *# 13.80 ± 10.50	*F*_1,38_ = 40.30, *p* < 0.001	+20.62 † +2.17
Back scratch test—Right hand (cm)	EG CG	0.54 ± 10.32 0.50 ± 10.00	2.15 ± 10.71 *# 0.45 ± 10.30	*F*_1,38_ = 30.10, *p* < 0.001	+74.88 † −11.11
Back scratch test—Left hand (cm)	EG CG	−4.49 ± 10.56 −4.40 ± 10.00	−3.83 ± 11.25 *# −4.50 ± 10.03	*F*_1,38_ = 25.15, *p* < 0.001	+17.23 † −2.22
Static balance					
Single limb stance test-Right leg (sec)	EG CG	48.54 ± 44.24 47.80 ± 45.00	107.14 ± 93.15 *# 50.50 ± 47.00	*F*_1,38_ = 45.40, *p* < 0.001	+54.69 † +5.35
Single limb stance test—Left leg (sec)	EG CG	64.75 ± 60.48 65.35 ± 59.50	117.22 ± 89.31 *# 64.45 ± 60.24	*F*_1,38_ = 35.50, *p* < 0.001	+44.76 † −1.40
Dynamic balance					
Time up and go test (sec)	EG CG	5.59 ± 0.62 5.57 ± 0.70	5.04 ± 0.59 *# 5.60 ± 0.65	*F*_1,38_ = 20.15, *p* < 0.001	−10.91 † +0.54

CG: control group; EG: exercise group. Where * *p* < 0.001, there is a statistically significant difference between baseline and final measurements in the EG; # *p* < 0.001 = statistically significant difference between the EG and CG in the final measurement; † *p* < 0.001 = statistically significant difference between the EG and CG in the percentage change.

**Table 7:** Strength parameters in exercise and control groups per measurement (Mean ± Standard deviation).

Variables	Group	Baseline Measurement	Final Measurement	Interaction Effect	Mean % Change
Maximum strength of lower limbs (kg)	EG CG	94.65 ± 38.25 93.67 ± 39.00	107.15 ± 35.46 *# 95.15 ± 33.00	*F*_1,38_ = 25.20, *p* < 0.001	+11.67 † +1.56
Maximum strength of trunk (kg)	EG CG	84.65 ± 34.23 85.35 ± 36.00	97.45 ± 31.32 *# 86.56 ± 30.50	*F*_1,38_ = 26.55, *p* < 0.001	+13.13 † +1.40
Maximum handgrip strength (kg)					
Right hand	EG CG	43.00 ± 13.74 42.55 ± 12.55	42.00 ± 14.56 41.50 ± 13.55	*F*_1,38_ = 2.55, *p* = 0.546	−2.38 −2.53
Left hand	EG CG	41.25 ± 14.59 41.50 ± 14.50	40.40 ± 13.69 40.55 ± 13.50	*F*_1,38_ = 2.46, *p* = 0.554	−2.10 −2.34

CG: control group; EG: exercise group. Where * *p* < 0.001, there is a statistically significant difference between baseline and final measurements in the EG; # *p* < 0.001 = statistically significant difference between the EG and CG in the final measurement; † *p* < 0.001 = statistically significant difference between the EG and CG in the percentage change.

**Table 8:** Aerobic capacity parameters in exercise and control groups per measurement (Mean ± Standard deviation).

Variables	Group	Baseline Measurement	Final Measurement	Interaction Effect	Mean % Change
HR before test (beats/min)	EG CG	78.35 ± 11.31 77.67 ± 12.00	73.15 ± 7.07 *# 78.00 ± 11.50	*F*_1,38_ = 25.35, *p* < 0.001	−7.11 † +0.42
HR at the end of the test (beats/min)	EG CG	137.54 ± 7.00 138.35 ± 8.60	130.62 ± 7.58 *# 137.66 ± 7.50	*F*_1,38_ = 20.65, *p* < 0.001	−5.30 † −0.50
Rate of perceived exertion (score)	EG CG	4.34 ± 2.25 4.50 ± 2.00	3.23 ± 2.01 *# 4.40 ± 1.55	*F*_1,38_ = 50.65, *p* < 0.001	−34.37 † −2.27
HR 1st min after test (beats/min)	EG CG	100.85 ± 9.95 99.50 ± 8.50	90.95 ± 9.64 *# 98.00 ± 9.00	*F*_1,38_ = 37.80, *p* < 0.001	−10.89 † −1.53

CG: control group; EG: exercise group; HR: heart rate. Where * *p* < 0.001, there is a statistically significant difference between baseline and final measurements in the EG; # *p* < 0.001 = statistically significant difference between the EG and CG in the final measurement; † *p* < 0.001 = statistically significant difference between the EG and CG in the percentage change.

**Table 9:** Enjoyment score (5-point scale) following the 3-month modified basketball exercise program.

Questions	Mean ± SD	Minimum	Maximum
Question 1: I enjoyed the activities of the program.	4.90 ± 0.31	4.00	5.00
Question 2: I thought that the program was interesting.	5.00 ± 0.00	5.00	5.00
Question 3: I thought that the time passed very quickly when I participated in the program.	4.95 ± 0.22	4.00	5.00
Question 4: When I participated in the program, I enjoyed it.	4.95 ± 0.22	4.00	5.00
Total score	4.95 ± 0.13	4.50	5.00

## Data Availability

Data are unavailable due to privacy or ethical restrictions.
